# Restriction of Cellular Plasticity of Differentiated Cells Mediated by Chromatin Modifiers, Transcription Factors and Protein Kinases

**DOI:** 10.1534/g3.119.400328

**Published:** 2019-05-14

**Authors:** Dylan P. Rahe, Oliver Hobert

**Affiliations:** Department of Biological Sciences, Howard Hughes Medical Institute, Columbia University, New York, NY

**Keywords:** C. elegans, plasticity, transcription factor, cellular fate

## Abstract

Ectopic expression of master regulatory transcription factors can reprogram the identity of specific cell types. The effectiveness of such induced cellular reprogramming is generally greatly reduced if the cellular substrates are fully differentiated cells. For example, in the nematode *C. elegans*, the ectopic expression of a neuronal identity-inducing transcription factor, CHE-1, can effectively induce CHE-1 target genes in immature cells but not in fully mature non-neuronal cells. To understand the molecular basis of this progressive restriction of cellular plasticity, we screened for *C. elegans* mutants in which ectopically expressed CHE-1 is able to induce neuronal effector genes in epidermal cells. We identified a ubiquitin hydrolase, *usp-48*, that restricts cellular plasticity with a notable cellular specificity. Even though we find *usp-48* to be very broadly expressed in all tissue types, *usp-48* null mutants specifically make epidermal cells susceptible to CHE-1-mediated activation of neuronal target genes. We screened for additional genes that allow epidermal cells to be at least partially reprogrammed by ectopic *che-1* expression and identified many additional proteins that restrict cellular plasticity of epidermal cells, including a chromatin-related factor (H3K79 methyltransferase, DOT-1.1), a transcription factor (nuclear hormone receptor NHR-48), two MAPK-type protein kinases (SEK-1 and PMK-1), a nuclear localized O-GlcNAc transferase (OGT-1) and a member of large family of nuclear proteins related to the Rb-associated LIN-8 chromatin factor. These findings provide novel insights into the control of cellular plasticity.

During the development of multicellular organisms, pluripotent cells differentiate into terminal fates through the adoption of various characteristics and features necessary for the proper functioning of the terminal cell type. Concomitant with differentiation, cells also progressively lose their original plasticity. Observations of the existence of cell fate restriction accompanied the first descriptions of pluripotency itself in the late 19^th^ century (Lensch and Mummery 2013). Classical embryology experiments involving the division of early newt and sea urchin embryos, in conjunction with subsequent embryonic tissue transplantation experiments by Hans Spemann and Hilde Mangold, were key in demonstrating both the plasticity of early embryonic cells as well as the progressive restriction of their fate as cells progressed through critical stages of development (Lensch and Mummery 2013). Pioneering somatic nuclear transplantation experiments further demonstrated the progressive loss of cell fate plasticity in differentiated cells (Briggs and King 1952; Gurdon 1960; Gurdon 1962). In these experiments, nuclei were removed from the endoderm of progressively later stage frog embryos and transplanted into enucleated eggs, with the number of fully developed tadpoles scored from each experiment. As the frog endodermal cells progressed further in development, they became increasingly restricted in their ability to return to a plastic state when transplanted.

In addition to embryological manipulation, the ectopic expression of critical cell-fate-specifying transcription factors (TFs) has also been used to study cell fate plasticity. The master regulator of muscle fate, MyoD, can induce the differentiation of multiple differentiated cell types into muscle cells (Weintraub *et al.* 1989). However, this TF is only able to do so in some cell types, while others are refractory to MyoD activity. In *C. elegans*, ectopic expression of the MyoD homolog *hlh-1* also results in the expression of muscle markers, but this activity drops off as development progresses (Fukushige and Krause 2005). Similarly, the endoderm specifying TFs END-1, END-3, ELT-2 and ELT-7 can drive expression of intestinal reporter constructs when ubiquitously expressed in early development (Sommermann *et al.* 2010). However, by the L1 stage, many fewer cells express the reporter. Using similar tools, the same lab later showed ELT*-7* to be capable of disrupting tissues in much later stages of development (Riddle *et al.* 2016). The transformations described are extensive but are restricted to a small subset of tissues in the animal, consistent with developmental fate restriction in most cell types.

Multiple factors have been shown to play a role in developmental fate restriction. In particular, genes involved in the deposition and maintenance of histone marks associated with transcriptional repression, namely H3K37 and H3K9 methylation, have been implicated. Removal of the PRC2 component *mes-2*/E(z) from the early embryo can extend the time period in which *hlh-1* or *end-1* ectopic expression results in aberrant adoption of muscle or endoderm fates, respectively (Yuzyuk *et al.* 2009). Our lab has studied this problem by investigating the effect of ectopic expression of the transcription factor CHE-1 (Tursun *et al.* 2011). *che-1* is normally expressed solely in the bilaterally symmetric ASE neuron pair (ASEL and ASER) and is required for the proper differentiation and function of this pair of chemosensory neurons (Chang *et al.* 2003; Uchida *et al.* 2003; Etchberger *et al.* 2007). In otherwise wild-type animals, ectopic expression of *che-1* is able to induce the expression of an ASER-reporter transgene (*gcy-5*::*gfp)* in a manner that is progressively restricted during development (Patel and Hobert 2017)(this work). Using this system, we found the histone chaperone *lin-53* to be critical in maintaining the cell fate restriction of germ cells (Tursun *et al.* 2011). Subsequent analysis found its role in the germline to be as part of complex with PRC2, as removal components of this complex (MES-2/E(z), MES-3, and MES-6/ESC) resulted in loss of fate restriction in the germline (Patel *et al.* 2012). Additionally, the FACT chromatin chaperone complex and the chromodomain-containing gene *mrg-1*/MRG15 have recently been demonstrated to be necessary to restrict cell fate transformations in the germline (Kolundzic *et al.* 2018; Hajduskova *et al.* 2019).

TFs responsible for proper differentiation also play important role in the restriction of cellular plasticity. In *C. elegans*, terminal selector-type TFs act in postmitotic neurons to specify the terminal identity of specific neuron classes (Hobert 2016). Using the ectopic CHE-1 expression assay, removal of a number of terminal selector TFs were found to permit CHE-1-mediated cellular reprogramming (Patel and Hobert 2017). This implies a dual role for the TFs responsible for fate specification in the first place — both in directing the adoption of a specific fate as well as restricting cellular plasticity. A more in depth characterization of a specific terminal selector TF, UNC-3, revealed a role of multiple chromatin remodeling factors in this process, including the H3K9 methyltransferases MET-2 and SET-25, suggesting a coordinated action of differentiation and cell fate restriction through master regulator TFs via histone modifying proteins (Patel and Hobert 2017).

Here we describe the results of two forward genetic screens designed to uncover novel factors involved in cell fate restriction. In the first, the same inducible CHE-1 ectopic expression system described above was used to identify multiple mutants that phenocopy *lin-53* and PRC2 components, namely a germline reprogramming phenotype. One mutant with a phenotype specific to the epidermis was isolated and cloned, *usp-48*, a ubiquitin hydrolase. In order to understand the mode of *usp-48* function, a second screen was performed to identify additional mutants that phenocopy *usp-48*. For this screen, we expressed *che-1* specifically in the adult epidermis using the promoter of *col-19*, an adult-specific collagen (Liu *et al.* 1995). 19 total mutants were isolated in this screen, 15 of which were cloned and correspond to alleles of seven genes, including a ubiquitin hydrolase, an H3K79 methyltransferase, a transcription factor, kinases, a glycosyltransferase, and a novel, nematode-specific gene.

## Materials and Methods

### Strains and transgenes

All strains were maintained using standard procedures (Brenner 1974), unless otherwise noted. For heat-shock experiments strains were grown at 20°, heat-shocked at 37° for 30min, and left overnight at 25°. A list of strains and transgenes is provided as Supplemental Table S1.

### Transgene generation

The *col-19* promoter (-844 to +12) was amplified from genomic N2 DNA using the following primers: 5′-GTACAGCATGCGCTTCCAAACGTCCCTATTAGG-3′, 5′-AGTCCCGGGGAGCTTGCCCATGTTGATGAACTG-3′. Primers contain *Sph*I and *Xma*I sites, which were used to ligate in to a pPD95.75-derived plasmid containing *che-1*::*2xFLAG* in place of GFP to generate pDR39. *col-19::che-1::2xFLAG* was PCR amplified using the following primers: 5′-GTGTGGAATTGTGAGCGGA-3′, 5′-AAGGGCCCGTACGGCCGACT-AGTAGG-3′ from pDR39, and injected into N2 at 5 ng/µL along with 5ng/µL pRF4 (*rol-6(su1006)*) and 130 ng/µL sheared OP50 genomic DNA. One extrachromosomal line was integrated by gamma irradiation and backcrossed 6x. Fosmid rescue lines were generated by injecting *Not*I-digested fosmids (for specific fosmids, see Suppl. Table S1), linearized *myo-2::rfp* vector, and sheared OP50 genomic DNA into mutants to be rescued.

### Mutagenesis

In the heat shock screen, synchronized late L4 larvae were mutagenized for four hours in 50mM ethyl methanesulfonate (EMS) in M9 media. Five F1s were plated to each plate, and each plate was heat shocked at 37° for one hour when F2s reached the L4/Young Adult stage. In the epidermis-specific screen, animals were mutagenized as before, but each generation was synchronized by alkaline bleach treatment. After bleaching, F1 embryos were distributed among 10 150mm NGM plates. When synchronized F2s reached adulthood, animals were washed extensively and sorted using a COPAS Biosorter for animals with high GFP expression. >300,000 F2 animals were sorted from each of the ten F1 plates, and Ecta animals were singled for further analysis.

### RNAi

Appropriate RNAi clones from Ahringer library (Kamath and Ahringer 2003) were grown O/N in LB+Carbenicillin, then 350 µL was plated onto NGM plates supplemented carbenicillin to 25 μg/ml and IPTG to 1mM. Once dried, 5-10 L4s were added. Phenotype was assessed in the following generation.

### CRISPR/Cas9 genome engineering

GFP insertions were engineered using co-CRISPR, as described in Kim *et al.* 2014. Homologous repair template for *usp-48* was a TOPO-cloned PCR-product from the GFP-tagged fosmid from the Transgeneomics Project (Sarov *et al.* 2012). GFP was inserted between the last codon and the stop codon of *usp-48*. The *ogt-1* repair template was generated by PCR fusion using the codon-optimized GFP sequence from Dickinson *et al.* 2015 and cloned into the pMiniT2 vector (NEB). The *dot-1.1::gfp* strain (*ot899*) was generatid using the SEC method (Dickinson et al. 2015).

### Single-molecule FISH

smFISH was performed using Custom StellarisTM FISH probes, purchased from Biosearch Technologies and staining was done according to the manufacturers protocol, with slight modifications. Briefly, animals were fixed in 10% formaldehyde in M9 for 30 min., then washed into 70% ethanol for 1-3 days at 4°. Animals were resuspended in 200 µl hybridization buffer (10% Formamide, 10% Dextran Sulfate, 1 mg/mL *E. coli* tRNA, 2 mg/mL Vanadyl ribonucleoside complex, 200 µg/mL BSA) to which probes were added to 5-10µM and incubated O/N at 37°. After hybridization, worms were washed in 2x SSC, 10% Formamide, incubated with 5 µg/mL DAPI, and mounted with antifade buffer for microscopy. Probes correspond to coding regions of *gcy-5* or *unc-10* cDNA (excluding UTRs).

### Microscopy

**Figure 1:**
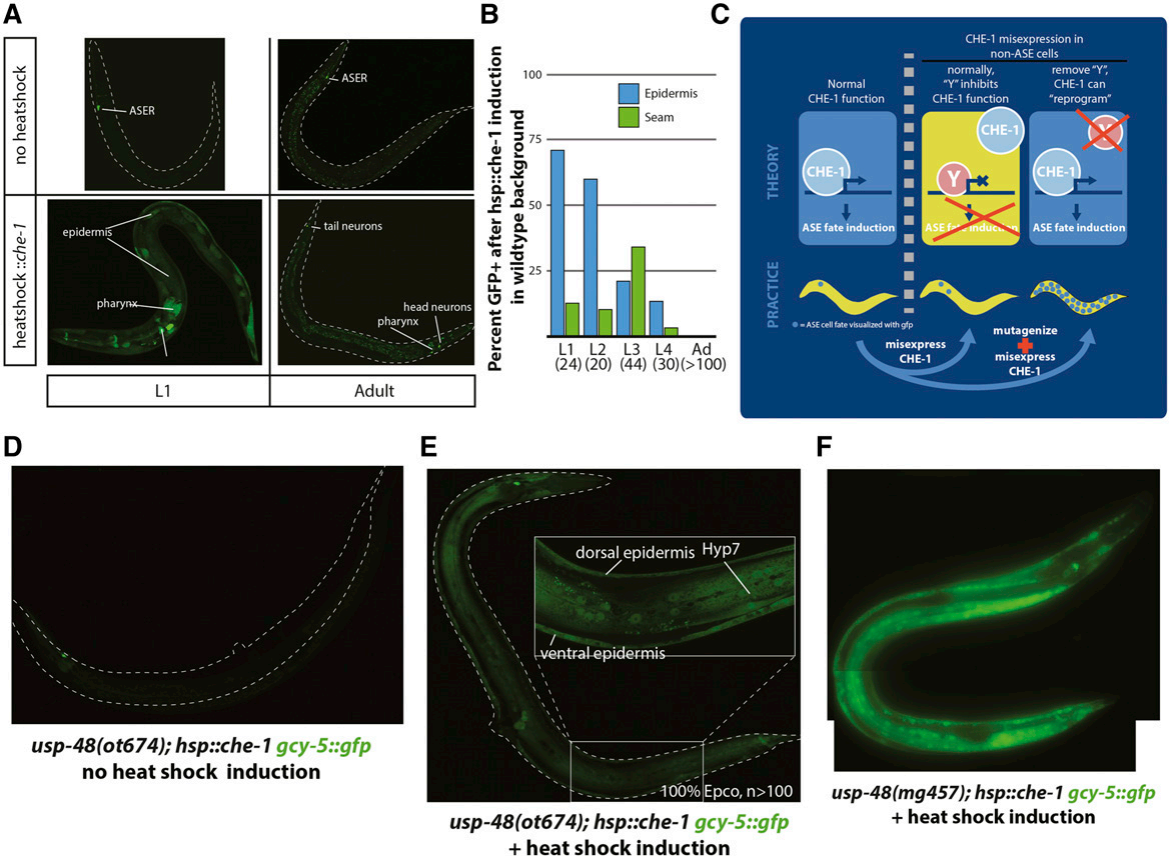
Basic parameters of the screening approach and isolation of the first mutant. A: Ectopic expression of *che-1* in an otherwise wild-type genetic background has diminished activity as development progresses. Without heatshock, *gcy-5*::*gfp* (*ntIs1*) is expressed solely in the ASER neuron. Heatshock induction of *che-1* during the early L1 stage results in broad activation of the *gyc-5* promoter, with GFP observed in the epidermis, pharnyx, multiple neurons, and other tissues. Heatshock during the adult stage is restricted to a few pharyngeal cells and extra neurons in the head and tail. B: Quantification of the results shown in panel A. Numbers below stage indicate animals scored. C: Outline for screening strategy and underlying hypothesis. *che-1* functions normally as a terminal selector transcription factor in the ASE neurons, and activates expression of multiple genes including *gcy-5*. If ectopically expressed in adult tissues, most tissues are refractory to activation of *gcy-5* target. It is hypothesized that some factor or factors, “Y”, prevent CHE-1 from activating *gcy-5* and other targets. This screen aims to identify the factor or factors “Y” by mutagenesis, with the resultant phenotype expected to be ectopic expression of *gcy-5* and other targets outside of what is normally seen in unmutagenized animals. D: Without ectopic induction of CHE-1, *usp-48(ot674)* animals express *gcy-5*::*gfp* only in ASER as in wild-type animals. E: After animal-wide heatshock induction of CHE-1, the *usp-48(ot674)* animals exhibit strong *gcy-5*::*gfp* expression specifically in the epidermis. F: *mg457* is another nonsense allele of *usp-48* and phenocopies *ot674*.

μManager, a plugin for ImageJ, was used for image acquisition and imageJ (Fiji) was used for processing on Figure 1F, Figure 3, Figure 6B&C, and Figure 9. Fig.S2 (Stuurman *et al.* 2007). All other images were taken on a LSM 880 Confocal microscope and processed using Zeiss Zen software.

### Tail epidermis ploidy measurements

Ploidy was assessed using DAPI staining (Lozano *et al.* 2006), with modifications. Specifically, day 1 adult hermaphrodite N2 animals were fixed in -20° DMF and washed immediately for four times with PBS-0.1% Triton-X100. During the second wash, 5 ng/mL DAPI was added and incubated for 30 min. at 4°, after which the final two washes were completed before mounting with Vectashield (Vector Labs). Animals were imaged using a Zeiss LSM 880 confocal microscope. Fluorescence intensity of individual nuclei was measured using ImageJ 2.0 software: Z-projection by sum-slices method, followed by freehand selection of nucleus, then Integrated Density using the measurement tool. Ploidy was estimated by normalizing to multiple neuronal nuclei from the tail region of each image.

### Wormsorter operation

COPAS Biosort (Union Biometrica) was used according to Doitsidou *et al.* 2008. Briefly, the Wormsorter was washed, then unmutagenized animals were recorded using green fluorescence (488nm exc. wavelength, GFP filter) and time-of-flight, a measure of the size of the sorted object. Objects of similar size as wild-type but with much higher levels of green fluorescence were sorted to plates, and a fluorescence dissecting scope was used to isolate individual Ecta mutants to new plates.

### Mapping-by-Sequencing

Mutants were mapped according to Doitsidou *et al.* 2010. Briefly, mutants are crossed to a strain (CB4856) that is polymorphic at known loci. F2 animals from this cross are then screened for the Ecta phenotype, and several (>15) are singled and allowed to grow to a large population size. These plates are then pooled together and genomic DNA extracted for sequencing.

### WGS library preparation, sequencing and bioinformatic analysis

*ot878*, *ot880*, *ot881*, *ot885*, *ot886*, and *ot887* were sequenced using 150 bp paired end reads on an Illumina HiSeq 2500 at BGI (Beijing Genomics Institute). All other sequencing libraries were prepared using Nextera tagmentation kits (Illumina) and sequenced using an Illumina NextSeq machine using either 75bp or 150bp read length. For the sequence analysis, FASTQ files were aligned to WS220 genome using *bwa-aln* (Li and Durbin 2009). Resulting bam files were sorted and indexed using *samtools* (Li *et al.* 2009). For SNV mapping, variant SNV alleles were called using the GATK “Best Practices” pipeline: duplicate reads are called with *picard* (http://broadinstitute.github.io/picard/), and GATK package was used both to recalibrate base scores according to known mapping variants (-T BaseRecalibrator) and call variants (-T HaplotypeCaller), both for known variant alleles (for mapping,–genotyping_mode GENOTYPE_GIVEN_ALLELES) and novel variants (for mutant analysis,–genotyping_mode DISCOVERY) (Depristo *et al.* 2011). A custom R script was used to plot the observed frequency of known SNVs *vs.* genome position to determine linkage. Allele frequency for each known variant was plotted independently for each chromosome *vs.* physical chromosome position, and a Loess regression line was fitted to the data. WGS without SNV mapping was performed using GATK without base recalibration. After a HaplotypeCaller run, variants were subtracted from other variants to create exclusive variant lists for each mutant using *bcftools isec* (Danecek *et al.* 2011; Narasimhan *et al.* 2016). With or without mapping, characterization of the effects of variants was performed using RefSeq gene structures and SnpEff (Cingolani *et al.* 2012). Scripts used in bioinformatics are available upon request.

### Data availability

Strains will be deposited at the Caenorhabditis Genetics Center (CGC) and are available upon request. Supplemental material available at FigShare: https://doi.org/10.25387/g3.8114237.

## Results and Discussion

### A genetic screen for cell fate plasticity reveals the ubiquitin hydrolase usp-48

We have previously established a powerful assay system to study the restriction of cellular plasticity (Tursun *et al.* 2011; Patel and Hobert 2017). Misexpression of the CHE-1 transcription factor, a master regulator of ASE neuron identity, under control of a ubiquitously expressed heat-shock promoter in embryonic or early larval stages is capable of inducing ASE identity features in other cell types, while misexpression in late larval or adult stages does not result in broad ectopic activation of ASE identity markers (Patel and Hobert 2017). We recapitulated these previously reported results by demonstrating that a short induction of CHE-1 expression from the *otIs305* array at early larval stages results in many tissues inducing ectopic expression of *gcy-5*, a direct target of *che-1* (Figure 1A,B). Heat shock at later stages show a progressive restriction of the induction of *gcy-5*::*gfp* in other tissues (Figure 1A,B). We sought to understand the underlying genetic basis of the progressive restriction of cellular plasticity by moving beyond our previous, candidate RNAi screens and instead screened in an unbiased manner for EMS-induced mutants in which ectopic induction of *che-1* expression in adult animals will permit induction of *che-1* target gene expression (schematized in Figure 1C).

A pilot, non-clonal mutagenesis screen identified several sterile mutants with ectopic *gcy-5*::*gfp* expression after *che-1* induction at adult stages, but we were not able isolate heterozygous parents to stably propagate these mutants. We therefore switched to a semi-clonal screen in which 5 F1s from a mutagenized P0 population were singled onto each screening plate. A total of 7302 haploid genomes were screened and 36 mutants were recovered. All mutants were sterile, and were maintained as heterozygotes. 35 of the 36 mutants displayed ectopic *gcy-5*::*gfp* in the germline. Since several such “germline-reprogammed” mutants had been described before (Tursun *et al.* 2011; Patel *et al.* 2012; Kolundzic *et al.* 2018; Hajduskova *et al.* 2019), no further analysis of this class of mutants was performed. We rather focused on the only mutant that exhibited a phenotype in somatic tissue, *ot674*. In *ot674* mutant animals, heat shock induction of *che-1* at any stage resulted in expression of *gcy-5*::*gfp* specifically in the epidermis of the animals (Figure 1E). We refer to this phenotype as the Ecta (Epidermal CHE-1 Target Activation) phenotype throughout this paper. The Ecta phenotype is specific to CHE-1 activity, as neither heat shock in the absence of *hsp*::*che-1* nor heat shock without the transgene results in ectopic expression of *gcy-5*::*gfp* in *ot674* mutants.

In an RNAi screen for chromatin factors whose depletion may permit *che-1* target gene induction (Tursun *et al.* 2011), we had observed, but not previously reported, that RNAi of one predicted chromatin factor, *usp-48*, resulted in an Ecta phenotype. We did not follow up this result due to the highly variable nature of the RNAi phenotype. However, after retrieval of the *ot674* mutant, we decided to sequence the *usp-48* locus in these animals through conventional Sanger sequencing and indeed found a nonsense mutation in the third exon of *usp-48*. The mutant was backcrossed and balanced with hT2, a chromosomal translocation between the first and third chromosome (McKim *et al.* 1988). In addition, a nonsense allele (*mg457*) previously isolated in the Ruvkun Lab for a different phenotype was obtained and was found to phenocopy *ot674* (Figure 1F). Yet another allele of *usp-48* was retrieved from another screen for the Ecta mutant phenotype, described in more detail below. The *usp-48* Ecta phenotype could be rescued with a wild-type copy of the gene, expressed under control of an epidermis-specific promoter. We conclude that *usp-48* is the causative mutation for the Ecta phenotype.

**Figure 2:**
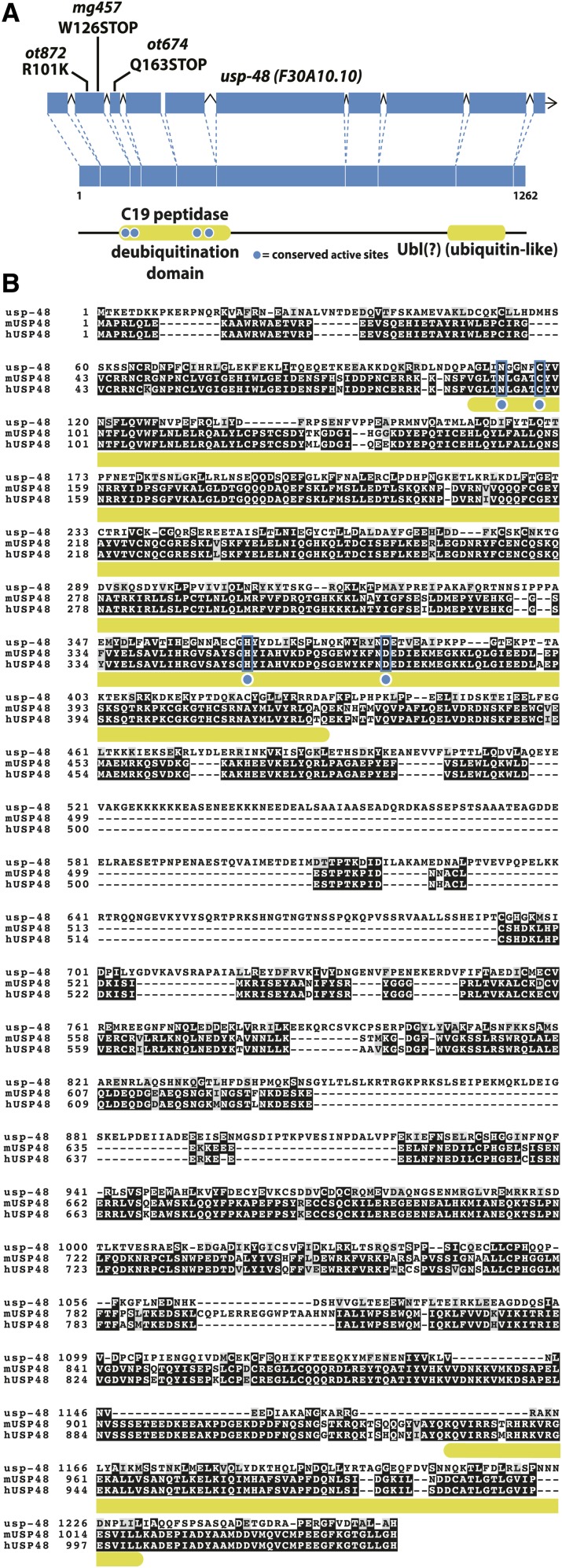
*usp-48* encodes a conserved ubiquitin hydrolase. A: USP-48 contains a conserved C19 peptidase domain as well as a ubiquitin-like (Ubl) domain at the C-terminus. Multiple alleles (*ot872*, *ot674*, and *mg457*) have been isolated and exhibit the Ecta phenotype. B: The C19 domain contains four catalytic residues, all of which are conserved in mouse and human orthologs (blue boxes), but the C-terminal Ubl domain is less well conserved in *C. elegans*, such that it is not recognized by domain searching algorithms (SMART, InterPROScan).

*usp-48* encodes the previously uncharacterized *C. elegans* ortholog of the human ubiquitin hydrolase USP48 (Komander *et al.* 2009) (Figure 2). Its function is not well understood, but recent studies on the mammalian homolog have suggested multiple roles. Two independent studies performing Chromatin Immunoprecipitation-Mass Spectrometry (ChIP-MS) on multiple histone marks coprecipitated USP48 with anti-H3K4me3, H3K4me1, H3K27ac, H3K79me2, and H3K36me3 antibodies (Engelen *et al.* 2015; Ji *et al.* 2015). All of these histone marks are associated with active transcription or promoter/enhancer activity (reviewed in (Gates *et al.* 2017)). This is notable because previously identified mutants with cell fate restriction phenotypes were mostly implicated in gene repression (Tursun *et al.* 2011; Patel *et al.* 2012; Kolundzic *et al.* 2018; Hajduskova *et al.* 2019). One exception is the *mes-4* gene, which restricts CHE-1 activity in the germline. *mes-4* deposits methylation on H3K36, a mark correlated with actively transcribed genes (Patel *et al.* 2012). H3K36 methylation plays a role in the proper distribution of the repressive H3K27 methylation mark (Gaydos *et al.* 2012; Patel *et al.* 2012).

### Assessing the effect of che-1 and usp-48 on endogenous gene expression

**Figure 3:**
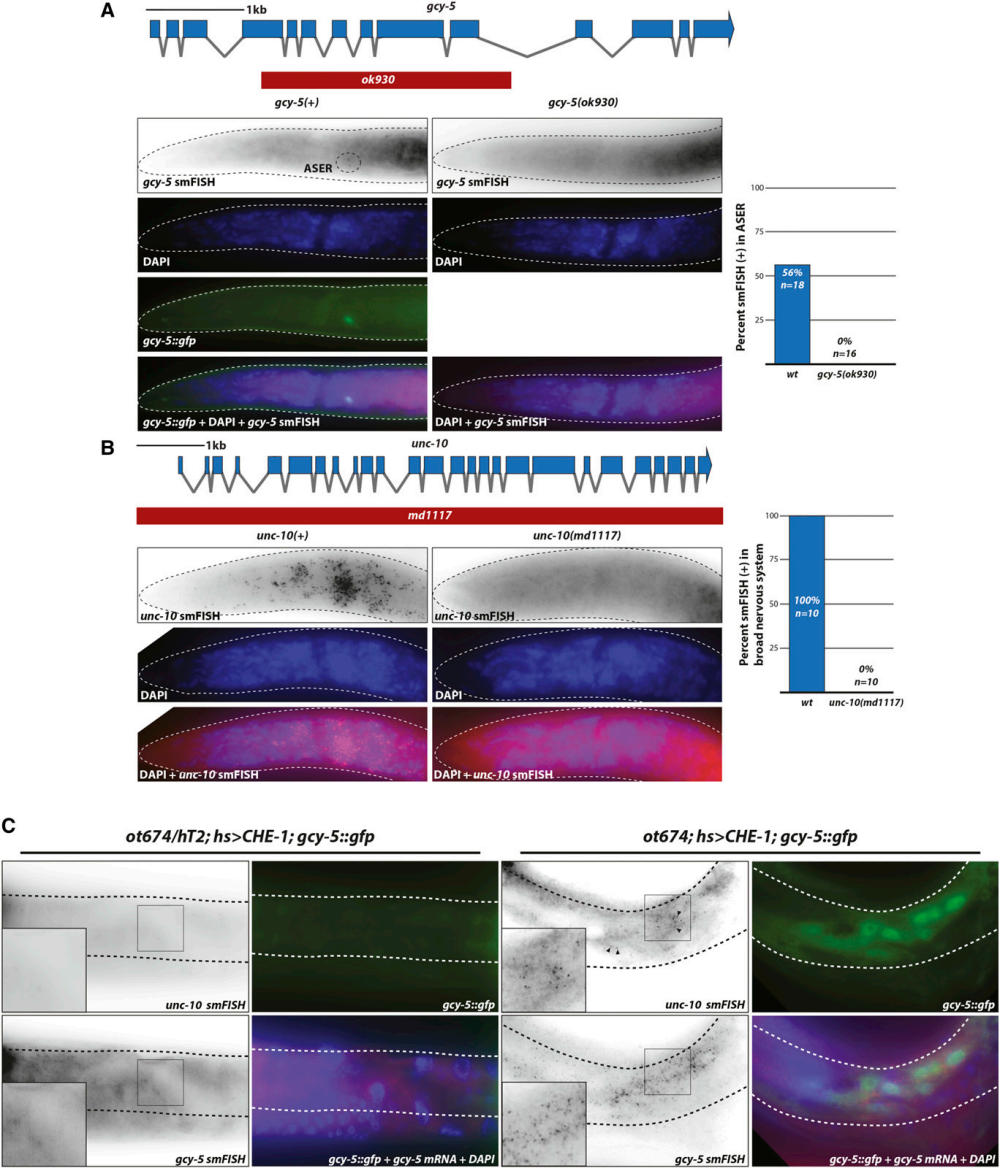
Induction of endogenous neuronal genes *gcy-5* and *unc-10* upon ectopic *che-1* expression in a *usp-48* mutant background. A,B: *gcy-5* (panel A) and *unc-10* (panel B) mRNA smFISH probes are gene specific, since staining of the respective deletion mutant animals revealed no puncta. Scoring of punctae is quantified in the right panels. C: Ectopic expression of *che-1* induces endogenous *gcy-5* and *unc-10* mRNA expression in the epidermis of *usp-48* mutants. After heat-shock inducation of *che-1* (*hs > che-1*), *gcy-5* and *unc-10* mRNA are induced in *usp-48* mutant epidermis (as assessed by smFISH), but not heterozygous controls.

We sought to assess the expression of CHE-1 targets in the epidermis of *usp-48* mutants by measuring endogenous mRNA using single-molecule fluorescence *in situ* hybridization (smFISH)(Raj *et al.* 2008), rather than transgenic reporters. In this method, fluorescence-labeled DNA oligonucleotides tiling an individual mRNA are hybridized in fixed animals, and individual mRNAs are thus observed as individual puncta. We generated smFISH probes to two ASE-expressed genes, *gcy-5* (a direct transcriptional target of CHE-1 protein) and *unc-10*, a pan-neuronally expressed synaptic protein (Koushika *et al.* 2001). Both genes are ectopically activated by *che-1* in a germ cell reprogramming paradigm (Tursun *et al.* 2011). We confirmed specificity of the smFISH probes by showing that fluorescent signals are not observed in *gcy-5* and *unc-10* deletion mutants, respectively, but detectable in wild-type animals with expected expression patterns (Figure 3A,B). In *usp-48(ot674)* mutants, but not heterozygous controls, punctate signals of the *unc-10* and *gcy-5* mRNAs were clearly visible within the epidermis after ectopic CHE-1 induction (Figure 3C). Thus, not only is a *gcy-5*-promoter-driven transgene activated, but also the endogenous locus itself, along with another marker of broad neuronal fate.

### Postdevelopmental requirement of usp-48 for restricting cellular plasticity

**Figure 4:**
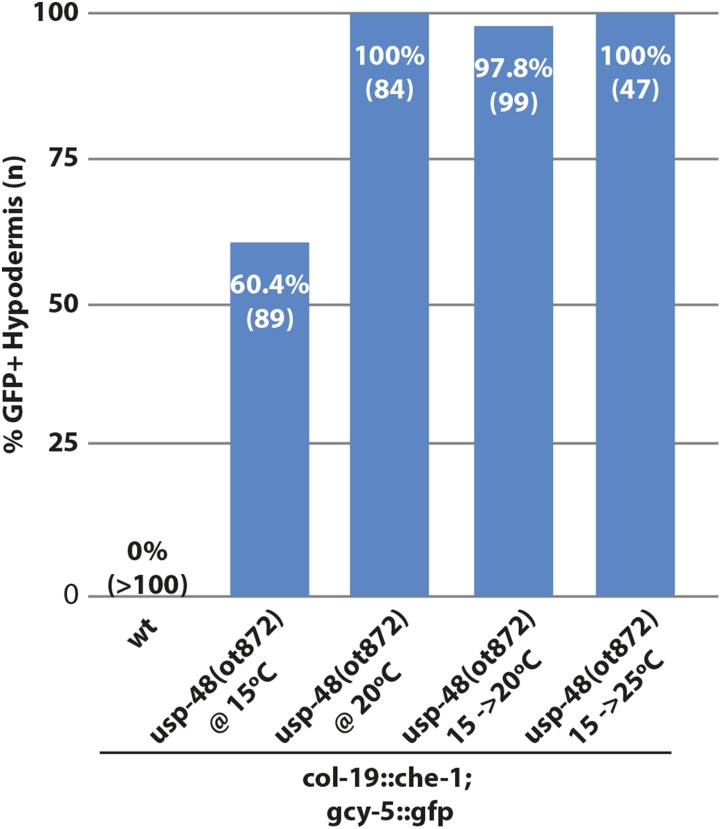
Assessment of postdevelopmental role of *usp-48* with *ot872*, a temperature sensitive allele. At 15°C, *usp-48(ot872)* is ∼60% Ecta, but at 20°C or higher the penetrance is ∼100%. When shifted at the L4 stage, before *che-1* expression begins, to either 20°C or 25°C, the animals are nearly completely penetrant Ecta.

A temperature-sensitive allele of *usp-48* that we retrieved from another screen, described below, allowed us to assess whether *usp-48* activity is required during the initial differentiation of the hypodermis or whether it is required in later, postdevelopmental stages to prevent *che-1* from inducing its targets genes. This temperature-sensitive allele, *ot872*, contained a point mutation in the second exon (Figure 2). At 25°, these animals exhibit some sterility and display 100% penetrant Ecta phenotype. At 20°, *ot872* animals are fertile but still display 100% penetrant Ecta phenotype. At 15°, these animals are fertile and show a significantly reduced penetrance of the Ecta phenotype (Figure 4). To access the postdevelopmental requirement of *usp-48*, we grew *ot872* animals at 15° and shifted them to 20° or 25° at the L4 stage. This temperature resulted in a 100% Ecta phenotype, reminiscent of animals raised continuously at 20 or 25° (Figure 4). These data suggest that USP-48 is required postdevelopmentally and acutely to restrict the plasticity of epidermal cells and prevent ectopic CHE-1 from being able to act on its targets.

### USP-48 protein is nuclear localized and very broadly expressed

**Figure 5:**
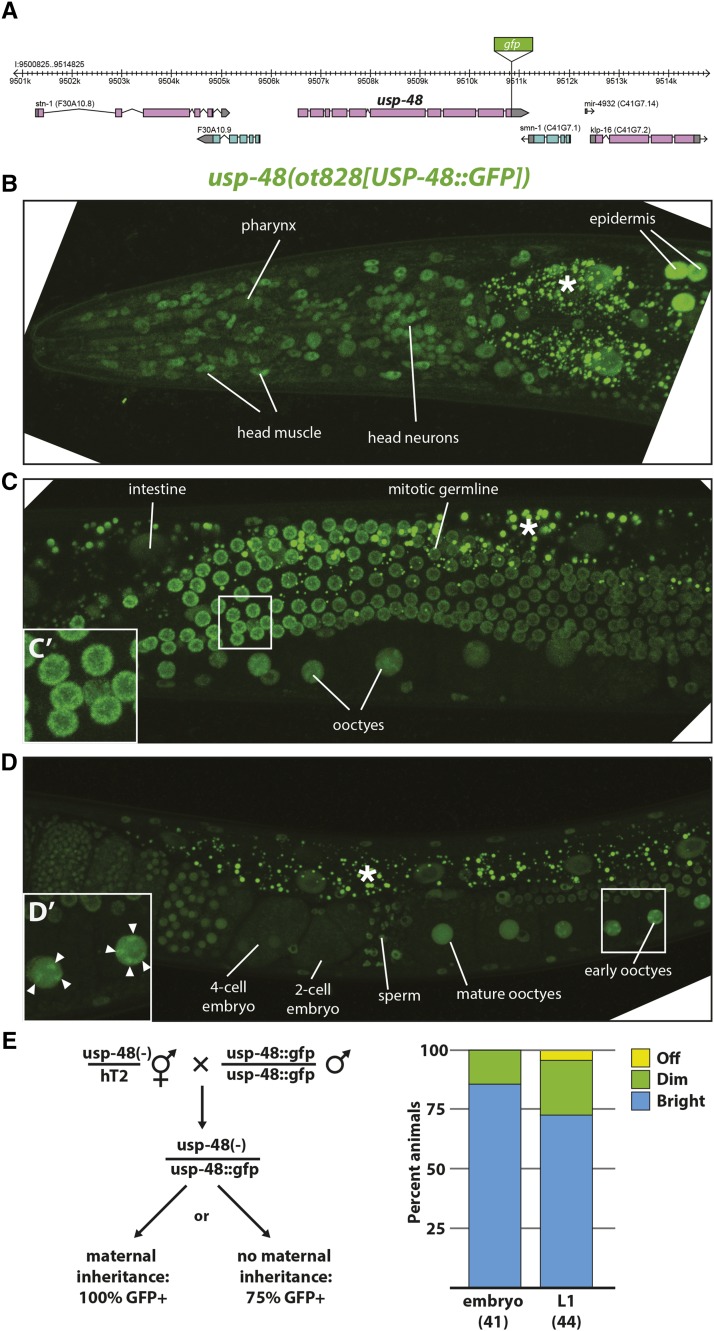
USP-48::GFP is broadly if not ubiquitously expressed, nuclear, and maternally inherited. A: Schematic of *usp-48* genomic locus into which GFP was inserted using CRISPR/Cas9. B: The CRISPR-generated GFP knock-in allele *ot828* exhibits broad nuclear expression throughout the anterior of the worm, if not ubiquitously, then very broadly. C. USP-48::GFP is expressed highly throughout the germline, and it is possible to see brighter regions inside developing oocytes, indicative of interactions with condensed chromosomes Inset (C') shows higher magnification of pachytene germ cells, in which bright USP-48::GFP regions resemble condensed chromatin. D. USP-48::GFP becomes diffuse in oocyte nuclei as they mature, then disappears from very early embryos, then reappears in the nucleus at the 4-cell stage, and is broad or ubiquitous from then on. Arrowheads in inset (D') indicate regions of brighter USP-48 accumulation, which resemble condensed chromosomes in mature oocytes. E: *usp-48*::*gfp* mRNA is maternally inherited. Left, schematic of cross used to determine maternal inheritance. Right, scoring of embryos from trans-heterozygous mother. 100% of progeny from *trans*-heterozygous *ot828/ot674* show at least some dim GFP expression in embryos, most of which persists into the L1 stage, indicating maternal inheritance of *usp-48* mRNA. (*) Indicates intestinal autofluorescence.

In order to further investigate possible roles of *usp-48*, an endogenous translational C-terminal GFP reporter was built using CRISPR/Cas9-mediated genome engineering (Figure 5A). The resultant *usp-48* allele exhibited nuclear GFP expression very broadly at all stages of development (Figure 5B-D). In pachytene germline cells and oocytes, clusters of brighter expression can be observed, consistent with chromatin binding (Figure 5C’,D’). The only observed cells not exhibiting nuclear expression were newly fertilized zygotes, until the 4- or 8-cell embryo, when nuclear expression can be observed again (Figure 5D). The absence of GFP signal in early embryos prompted us to ask if *usp-48* mRNA was maternally inherited. To this end, progeny from a *trans*-heterozygote of this CRISPR allele and *ot674* were generated. If *usp-48* is maternally inherited, 100% of resultant embryos should display GFP expression. This was the case, with fluorescence persisting to a lesser extent into the L1 stage. Thus, there exists maternal contribution of the mRNA despite loss of GFP signal in the early embryonic nuclei (Figure 5E).

**Figure 6:**
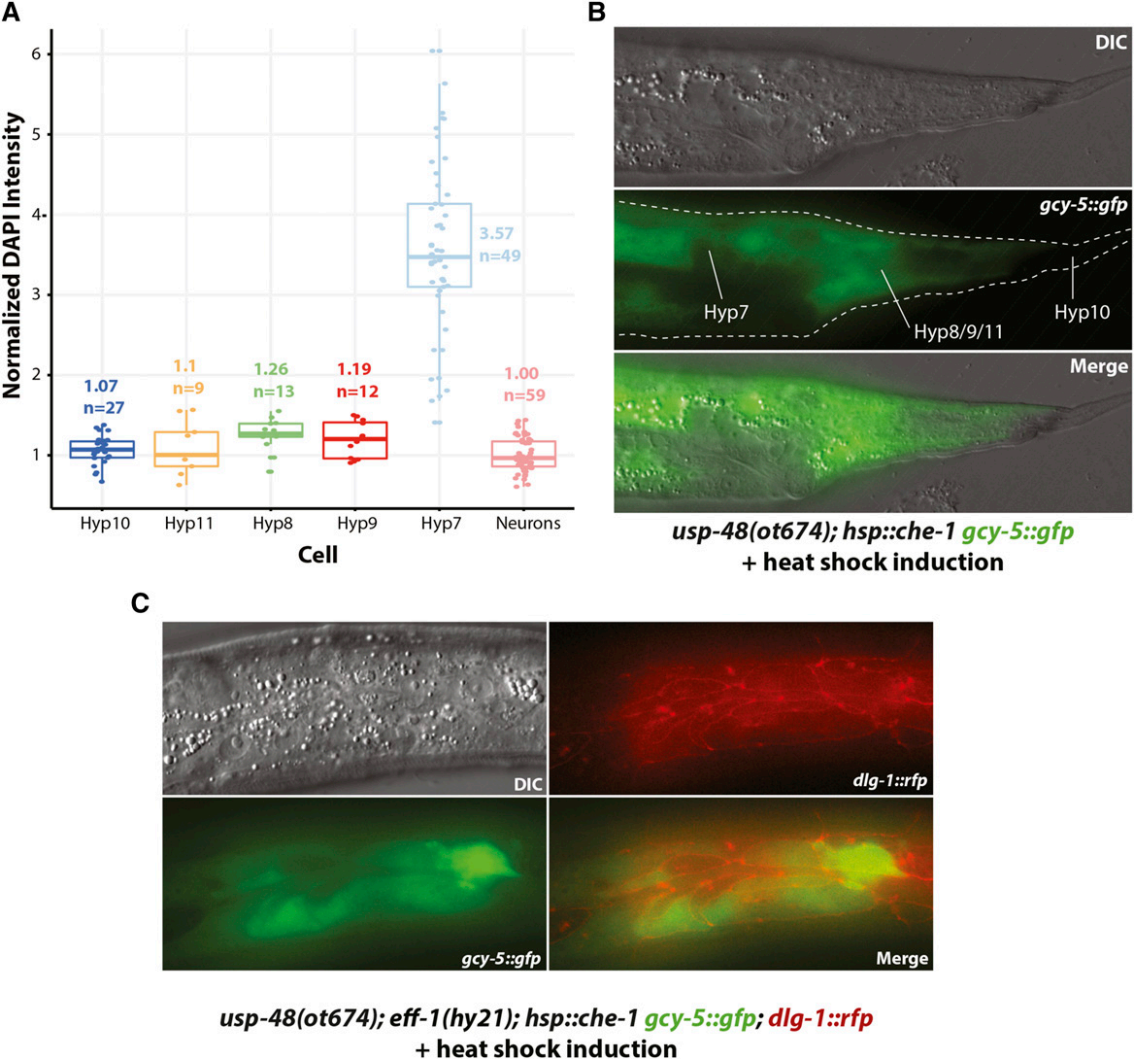
Specific epidermal features not required for cellular plasticity. A,B: Polyploidy of epidermal cells is not the reason why *che-1* can induce *gcy-5* in *usp-48* mutants. A: Assessment of ploidy of epidermal cells. Hedgecock and White had discovered polyploidy of hyp7 cells (Hedgecock and White 1985) but the assessment of ploidy of tail hypdermal cells was not reported in their study and is shown here. See Methods for details. B: Tail epidermal cells exhibit *gcy-5*::*gfp* expression after heatshock induction of CHE-1 in *usp-48(ot674)* animals. C: Epidermal cell fusion is not required for susceptibility of *gcy-5*::*gfp* expression after heatshock induction of CHE-1, as assessed in *eff-1*; *usp-48(ot674)* mutants.

The expression of *usp-48* in all cell types, combined with the ubiquitous induction of *che-1* in our protocol, raises the intriguing question as to why it is only the epidermis that becomes susceptible to *che-1*-misexpression. *usp-48* does not appear to display selective defects in epidermal differentiation. At the young adult stage, when *usp-48* mutants display the Ecta phenotype, the epidermis of *usp-48* mutants looks superficially normal by light microscopy and animals are not Dumpy or Blistered (common phenotypes associated with epidermal defects) and have normal adult alae. Moreover, we examined the expression of two collagens, terminal markers of epidermal identity, *dpy-7* and *col-93*, and found no effects of *usp-48* on the expression of these genes. One feature of many *C. elegans* epidermal cells that make their nuclear biology different from other cells in the worms is that epidermal nuclei continue to replicate their DNA during larval development and adulthood, a process known as endoreduplication (Hedgecock and White 1985). One could envision that this could lead epidermal chromatin to be more “susceptible” to *usp-48* and *che-1*-dependent gene expression changes, compared to other tissue types. However, we can rule out this possibility, because we observe *che-1* target activation in *usp-48* mutants upon *che-1* misexpression in non-syncytial epidermal cells of the tail, which do not endoreduplicate (Figure 6A,B). Other cell types that endoreduplicate (*e.g.*, the intestine) also show no *gcy-5* induction, corroborating that endoreduplication alone cannot explain the phenotype. We furthermore note that since tail epidermal cells do not fuse with other epidermal cells, as do the epidermal cells derived from dividing seam cells during larval development (Hedgecock and White 1985), we can also rule out that processes related to cell fusion may explain the particular sensitivity of epidermal cells to *che-1*-dependent, ectopic gene activation in *usp-48* mutants. Consistent with this notion, in *eff-1* mutants, where all epidermal cells fail to fuse (Mohler *et al.* 2002), *che-1* is still able to activate *gcy-5:gfp* in *usp-48* mutants (Figure 6C). One possibility that could explain the epidermal specificity and which we cannot completely rule out is that the expression levels achieved by the heat shock promoter may be higher in the epidermis than in other tissues.

### A different screen that probes specifically for epidermal cell fate plasticity reveals a large set of plasticity restriction mutants

**Figure 7:**
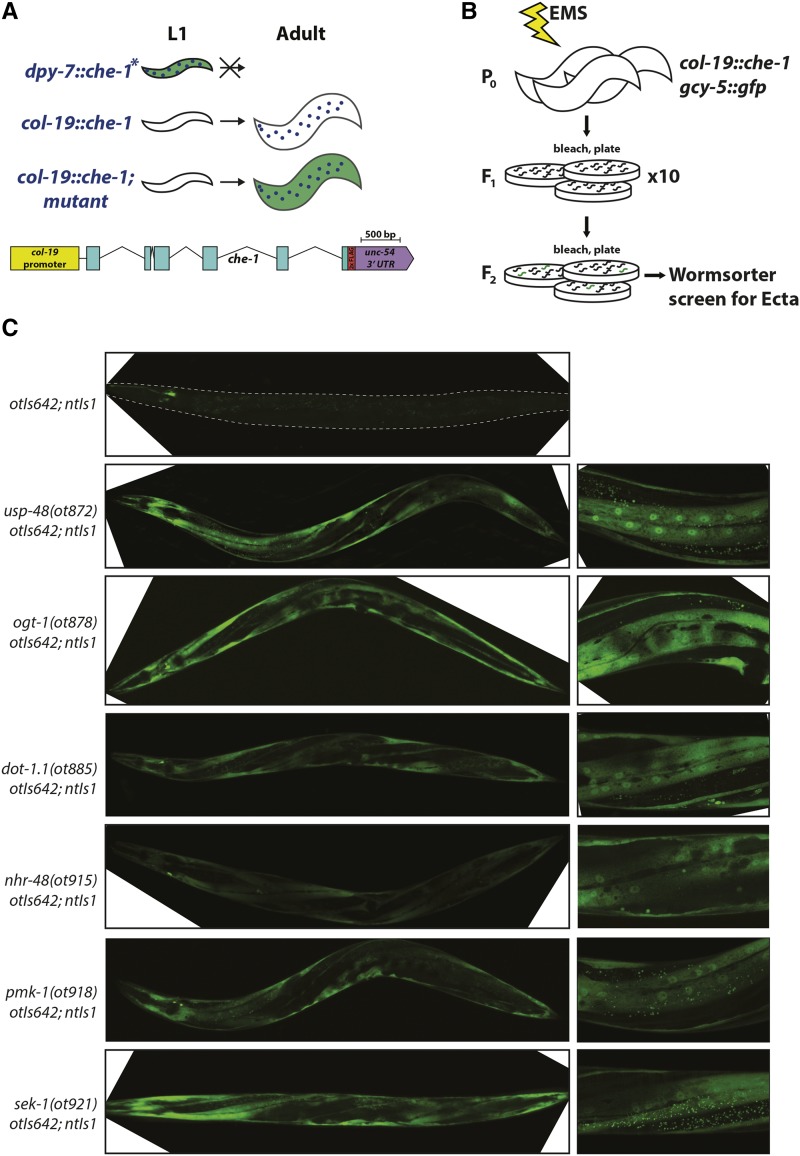
Screening for Ecta mutants using *col-19*::*che-1* misexpression. A: Worms expressing *che-1* under the control of an early epidermal promoter are not viable, displaying *gcy-5*::*gfp* fluorescence in the epidermis at the L1 stage (no stable lines could be established)(Patel and Hobert 2017), whereas animals with a *col-19*::*che-1* transgene *(otIs642)* are fertile and do not express *gcy-5*::*gfp* in the epidermis at any stage (panel C). In this screen, mutants are isolated in which *gcy-5*::*gfp* is seen in the epidermis at the adult stage, when che-1 is expressed. Included is a schematic of *col-19*::*che-1* transgene. B: P0 animals are mutagenized, F1s are split among 10 plates, and F2s are screened using the COPAS Biosort “Wormsorter” for the Ecta phenotype. C: Mutants identified in this screen. Representative micrographs of mutants isolated in the screen. Top panel shows *otIs642*; *ntIs1* alone, where no expression of *gcy-5*::*gfp* (*ntIs1*) is observed in the epidermis, despite *che-1* expression. Panels on the right are higher magnification micrographs of the panels on the left. In all mutant strains strong *gcy-5*::*gfp* expression is observed in the epidermis.

In order to perhaps better understand *usp-48* function, we set out to screen for mutants that phenocopy the Ecta phenotype of *usp-48*. The isolation of so many sterile mutants (that we did not further pursue, as mentioned above), made us concerned that our heat-shock based approach to induce *che-1* expression may make animals unnecessarily sick. To avoid this issue, we designed a different screening approach. We constructed a new transgene (*otIs642*) that expresses *che-1* under the control of the promoter of *col-19*, which drives expression from the L4/Adult molt onwards specifically in the epidermis (Cox and Hirsh 1985; Liu *et al.* 1995). In contrast to a transgene that expresses *che-1* throughout all developmental stages in the epidermis (*dpy-7^prom^*::*che-1*) and which results in the ectopic induction of *gcy-5*::*gfp* expression in the epidermis (Patel and Hobert 2017), this transgene does not induce *gcy-5*::*gfp* expression (Figure 7A, C). The different effects of these two transgenes corroborate the phenomenon of plasticity restriction in the adult epidermis. Using this *col-19^prom^*::*che-1* transgene, we conducted a non-clonal screen with the aid of a Wormsorter, a FACS machine specialized for sorting of entire animals (Doitsidou *et al.* 2008). In order to reduce the possibility of isolating siblings from the same mutant F1, we separated F1s onto ten different plates. Thus, individual Ecta animals from different screening plates are assured to be independent alleles (Figure 7B). This approach indeed yielded a large number of completely fertile mutants. All but one plate contained multiple Ecta mutants that were fertile and bred true. Of those that bred true, we were able to maintain 23 strains that were both highly penetrant and expressive.

The 23 mutant strains were first screened for mutations in the *usp-48* locus by Sanger sequencing. One of them turned out to be the temperature-sensitive *usp-48* allele, *ot872*, that we already described above. 14 strains that harbored no mutation in *usp-48* were selected for whole genome sequencing (WGS) in combination with SNP-based mapping, using a procedure we previously described (Doitsidou *et al.* 2010). Each allele was mapped to a specific region through such linkage analysis, and through a combination of complementation testing, transgene rescue, and/or RNAi phenocopy experiments we were able to map nine of these alleles to five distinct additional genes, as described below. The remaining four alleles have been mapped to a chromosomal region but remain uncloned.

**Figure 8:**
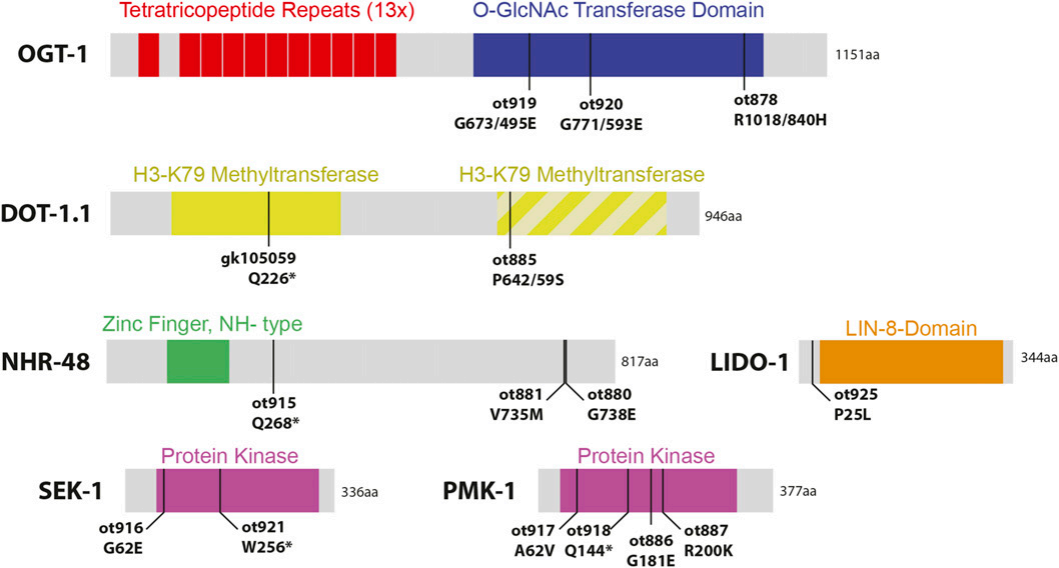
Molecular identity of Ecta mutants. Schematic of gene products identified in this screen. All sizes are approximately proportional. Hatched second domain of DOT-1.1 is due to only being recognized by some algorithms, and having poor alignment indicates poor homology with other H3K79 methyltransferase domains.

The remaining eight mutant strains retrieved from the screen were subsequently whole genome-sequenced without any mapping, which revealed (a) two additional alleles of the genes that we mapped in parallel, (b) four strains were revealed to be siblings of already identified mutations, and (c) two alleles of a gene (*sek-1)* whose function is intimately related to one of the genes that we first cloned (*pmk-1*; explained further below). In summary, 23 strains were analyzed, of which 19 were confirmed to be independent alleles, 15 of which correspond to seven genes that we molecularly identified, and four mutants remain uncloned. Relevant alleles are summarized in Table 1 and representative micrographs of these mutants are shown in Figure 7C. Cloning information is summarized in Table 2, mapping information and gene structures and locations of mutations are shown in Figure 8. The following genes were identified by this screen.

**Table 1:** Summary of Ecta mutants isolated from screens

Locus	alleles
*usp-48*	*ot674*, *ot872*
*dot-1.1*	*ot885*
*ogt-1*	*ot919*, *ot920*, *ot878*
*lido-1*	*ot929*
*pmk-1*	*ot917*, *ot918*, *ot886*, *ot887*
*sek-1*	*ot916*, *ot921*
*nhr-48*	*ot915*, *ot880*, *ot881*
	*ecta(ot926) X ^1^*
	*ecta(ot927) I ^1^*
	*ecta(ot928) II^1^*
	*ecta(ot930) X ^1^*

1 Uncloned. Mapped onto chromosomes via Hawaian-SNP mapping.

**Table 2:** Effects of epidermal, adult misexpression of CHE-1 in wildtype and mutant backgrounds. Representative mutants and controls scored. * Grown at 20°

Genotype (all with *ntIs1* transgene)	% animals with Ecta phenotype	n
*otIs642 [col-19p*::*che-1]*	0	>100
*usp-48(RNAi)*; *otIs642*	68.4	73
*usp-48(ot872)*; otIs642*	98.7	78
*ogt-1(ot878)*	0	>100
*ogt-1(ot878);otIs642*	84.3	108
*ogt-1(RNAi)*; *otIs642*	68.3	41
*ogt-1(ok430)*; *otIs642*	85.1	67
*ogt-1(ot878);otIs642*; *ex[ogt-1 fosmid]*	0	37
*dot-1.1(ot885)*	0	50
*dot-1.1(ot885)*; *otIs642*	98.1	53
*dot-1.1(RNAi)*; *otIs642*	39.6	101
*nhr-48(ot915)*	0	83
*nhr-48(ot915)*; *otIs642*	93.4	91
*nhr-48(ot881)*; *otIs642*	96.2	52
*nhr-48(ot881)*; *otIs642*; *ex[nhr-48 fosmid]*	12.9	62
*pmk-1(ot918)*	0	95
*pmk-1(ot918)*; *otIs642*	100	67
*pmk-1(km25)*; *otIs642*	92.4	79
*sek-1(ot921)*	0	63
*sek-1(ot921)*; *otIs642*	98.4	64
*lido-1(ot929)*	0	84
*lido-1(ot929)*; *otIs642*	80.1	108
*lido-1(RNAi)*; *otIs642*	99	84

### dot-1.1/DOT1L, an H3K79 methyltransferase, restricts cellular plasticity in the epidermis

**Figure 9:**
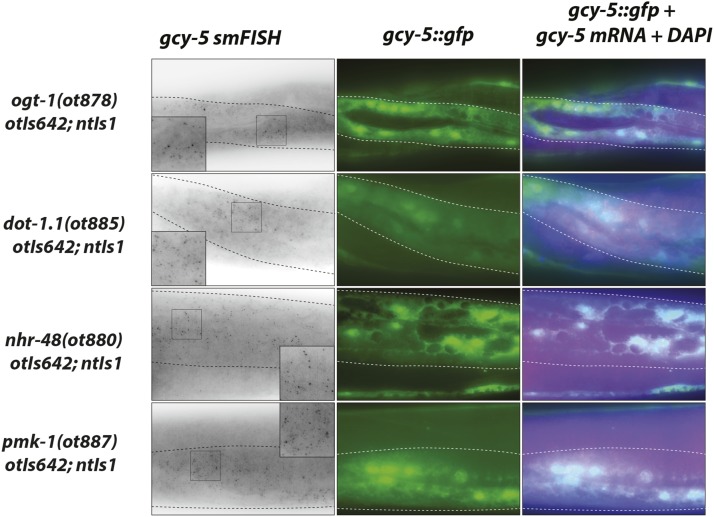
Ectopic expression of *che-1* induces endogenous *gcy-5* and *unc-10* mRNA expression in several Ecta mutants. *ogt-1*, *dot-1.1*, *nhr-48*, and *pmk-1* mutants exhibit ectopic *gcy-5* mRNA expression in the epidermis in addition to *gcy-5*::*gfp* expression, as assessed by smFISH analysis. Insets are zoomed in regions indicated in panel. Probe specificity is validated in Figure 3 A, B.

The *ot885* allele was mapped to the left arm of chromosome I and found to contain a missense mutation in *dot-1.1* which codes for one of five *C. elegans* orthologs of the phylogenetically conserved H3K79 histone methyltransferase DOT1 (Feng *et al.* 2002). An available nonsense mutation and RNAi against *dot-1.1* phenocopied the defects observed in *ot885* animals (Table 2). As with *usp-48*, we find that the ectopic expression of the *gcy-5* reporter transgene after *che-1* induction in *dot-1.1* mutants is an accurate reflection of endogenous *gcy-5* transcription since we observed endogenous *gcy-5* induction by smFISH analysis (Figure 9).

The retrieval of *dot-1.1* from this screen and its phenotypic similarity to *usp-48* mutants is notable because in vertebrates, chromatin immunoprecipitation with antibodies raised against the H3K79 mark deposited by DOT-1.1 pulls down vertebrate USP48 protein (Engelen *et al.* 2015; Ji *et al.* 2015). *dot-1.1/DOT1L* is a unique methyltransferase both in that it does not methylate a histone tail, but rather a residue residing within the core globular domain of the histone H3, and that it uses a domain other than SET to catalyze this activity (Wood *et al.* 2018). Despite its original characterization as being required for silencing of telomeres, H3K79 methylation by Dot1 and its homologs is most closely associated with actively transcribed genes (Wood *et al.* 2018), as is USP48 by virtue of its physical association with histone marks associated with active genes (Engelen *et al.* 2015; Ji *et al.* 2015).

### ogt-1*/*OGT, the sole intracellular O-GlcNAc Transferase, also restricts cellular plasticity in the epidermis

Another mutant allele causing an Ecta phenotype, *ot878* (Figure 7C), mapped to chromosome III and contained a missense mutation in *ogt-1* (Figure 8). A null allele of this gene as well as RNAi resulted in the same Ecta phenotype. Two additional missense alleles of *ogt-1* (Figure 8, Table 1) were identified by sequencing two independently isolated mutants in the screen. As with *usp-48* and *dot-1.1*, we find that the ectopic expression of the *gcy-5* reporter transgene upon *che-1* expression in *ogt-1* mutants is an accurate reflection of endogenous *gcy-5* transcription since we observed endogenous *gcy-5* induction by smFISH analysis (Figure 9).

*ogt-1* encodes the sole *C.elegans* ortholog of O-GlcNAc Transferase (OGT), an enzyme responsible for the addition of a single N-acetyl Glucosamine (GlcNAc) carbohydrate moiety to a serine or threonine hydroxyl group (reviewed in (Levine and Walker 2016)). While the vast majority of glycosylation occurs in the endoplasmic reticulum or Golgi, OGT is the sole example of intracellular glycosylation known. OGT has thousands of cytoplasmic and nuclear targets, and is thought to achieve specificity by protein-protein interactions with non-catalytic tetratricopeptide repeats that are present in the N-terminal half of the protein. OGT mutants are lethal in mammals, zebrafish, and flies, but, surprisingly, *ogt-1* deletion alleles are viable in *C. elegans* (Hanover *et al.* 2005)(this study). OGT in Drosophila was originally identified as a Polycomb-group gene *super sex combs*, linking its activity to transcriptional regulation (Sinclair *et al.* 2009). Some evidence has been reported for the existence of O-GlcNAc modifications of histones H2A, H2B, H3 and H4 (Sakabe *et al.* 2010; Zhang *et al.* 2011). However, other studies have found no evidence for this modification on histones (Gagnon *et al.* 2015). What is less controversial is the existence of multiple TFs and even Pol II as substrates of OGT (Nagel and Ball 2014; Lewis *et al.* 2016). Either indirectly through TFs or directly, OGT and O-GlcNAc has been found to associate with chromatin, mainly upstream of highly active promoters in both worms and mammals (Love *et al.* 2010; Deplus *et al.* 2013). Hence, to the extent that OGT has been shown to associate with chromatin, it is mainly correlated with active transcription, as observed for USP48 and DOT1L.

### pmk-1/p38α and sek-1/MKK3/6, members of a conserved stress-signaling pathway, restrict cellular plasticity in the epidermis

Mapping to the same region of chromosome IV, the Ecta mutant alleles *ot886* and *ot887* contained missense mutations in the *pmk-1* locus. This gene encodes the *C. elegans* ortholog of p38-alpha MAPK, a gene involved in a number of distinct processes in *C. elegans*, including innate immunity (Kim *et al.* 2002)(Figure 8). Two additional mutant strains with an Ecta phenotype, in which we had not mapped the phenotype-causing mutation, each contain a mutation in the *pmk-1* locus (one missense and one nonsense allele) (Table 1, Figure 8). We tested a known null allele of *pmk-1* and found it to also cause the Ecta phenotype, as does *pmk-1(RNAi)* (Table 2). As with *usp-48*, *dot-1.1* and *ogt-1*, we find that the ectopic expression of the *gcy-5* reporter transgene upon *che-1* expression in *pmk-1* mutants is an accurate reflection of endogenous *gcy-5* transcription since we observed endogenous *gcy-5* induction by smFISH analysis (Figure 9).

The PMK-1 kinase is activated by the MAP kinase kinase SEK-1 (Kim *et al.* 2002) and we found that two strains in our mutant Ecta collection contained a mutation in the *sek-1* locus (Table 1, Figure 8). p38 MAP kinases have been shown to associate with chromatin (Ferreiro *et al.* 2010; Segalés *et al.* 2016). The localization of these factors to chromatin appears to be mediated through interactions with their TF targets, though additional targets have been identified linking p38 to transcriptional elongation, chromatin remodeling via SWI/SNF, and the repression of KMT1A-mediated H3K9 methylation (Simone *et al.* 2004; Ferreiro *et al.* 2010; Kumari *et al.* 2013). Given our retrieval of both *pmk-1* and *ogt-1* mutants, it is particularly interesting to note that p38 has been shown to activate OGT during glucose deprivation, and while p38 does not phosphorylate OGT, they physically interact with one another in neuroblastoma cells (Cheung and Hart 2008). Both proteins have been subjected to ChIP analysis and characterized as interacting with a suite of genes that are, in the cell types analyzed, highly transcribed (Deplus *et al.* 2013; Segalés *et al.* 2016). This matches the theme of the USP48 and DOT1L association with actively transcribed chromatin, as discussed above.

### lido-1 is a member of a large Caenorhabditis gene family

**Figure 10:**
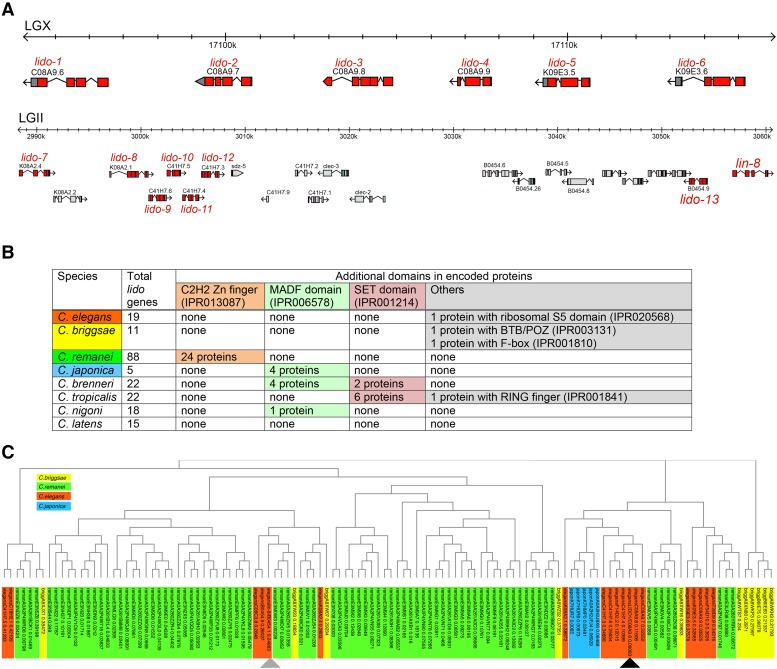
Lido protein family. A: Two genomic clusters of *C. elegans lido* genes on LGII and LGX. B: Domain organization of LIDO proteins. C: Phylogenetic tree of nematode LIDO proteins. Gray and black tringles indicate *lin-8* and *lido-1*, respectively.

One of the mutant allele was mapped to the right arm of LGX and found to harbor a mutation in a gene, C08A9.6, which we named *lido-1* (for LIN-8 domain containing). The causative nature of the mutant allele is suggested by phenocopying the effect of this mutation by RNAi against *lido-1* (Table 2). *lido-1* is one of 16 genes in the *C.elegans* genome whose protein shares a central domain of homology (Interpro accession number IPR005020) with the previously described LIN-8 protein (Davison *et al.* 2005)(Figure 10). We have named all other genes that contain this domain of homology *lido* as well. In most of these proteins this domain encompasses almost the entire protein. Two additional *C. elegans* genes are likely pseudogenes with premature stop codons; without such codon their protein products would also contain a LIN-8 domain. Most *lido* genes can be found in two clusters, one on the X chromosome (containing 6 directly adjacent *lido* genes) and one on the second chromosome (containing 7 *lido* genes plus *lin-8*) (Figure 10A).

Intriguingly, by inspecting other genome sequences, we find that other *Caenorhabditis* species have significantly expanded or retracted their complement of *lido* genes. *C. remanei* encodes 88 *lido genes*, while *C. japonica* only contains only 5 *lido* genes (Figure 10B). Equally intriguingly, in other *Caenorhabditis* species, the encoded proteins have acquired additional domains, most of them involved directly in DNA or chromatin-related activities. For example, 21 of the *C. remanei* LIDO proteins have C2H2 Zn finger DNA binding domains, a total of 9 *C. brenneri*, *C. japonica* or *C. nigoni* proteins have MADF DNA binding domains and a total of 8 *C. brenneri* or *C. tropicalis* proteins have histone methylating SET domains. There are few easily recognizable one-to-one orthologs in the LIN-8 domain proteins in the different *Caenorhabditis* species (Figure 10C), indicating that these proteins have independently expanded and contracted after speciation.

Outside the *Caenorhabditiae*, only two species contain this domain of homology, a protein from a single Cyanobacteria species (*Moorea producens*) and a single protein from the ciliate *Pseudocohnilembus persalinus*. According to the Interpro database, the LIN-8 domain of the ciliate protein displays also some homology to a SET domain, suggesting that the LIN-8 domain and the SET domain may share some evolutionary relationship. The homology of LIDO-1 to LIN-8, a class A SynMuv gene, is a further indication of a function of LIDO-1 in chromatin biology. Synthetic multivulva (SynMuv) genes were identified as being redundantly required (Synthetic) for the inhibition of *let-60*/Ras signaling during the formation of the vulva (Fay and Yochem 2007). SynMuv mutants in each class correspond to groups of interacting genes, and of the two major classes, the SynMuvB class contains homologs of DP, E2F, and Rb, several of which have been suggested to form various transcriptionally repressive complexes, including the DRM/DREAM and NuRD complexes (Fay and Yochem 2007). SynMuvA class genes, however, which include *lin-15A*, *lin-8*, *lin-38* and *lin-56*, encode novel nuclear proteins with no non-nematode orthologs (Davison *et al.* 2005; Fay and Yochem 2007; Davison *et al.* 2011). LIN-8 has been shown to physically interact with the LIN-35/Rb transcriptional repressor (a SynMuvB gene)(Davison *et al.* 2005).

While the specific function of the LIN-8 domain is not known, it is possible that LIDO-1, similar to LIN-8, interacts with other nuclear genes involved in transcriptional repression, or possibly LIN-35/Rb itself, given the extensive homology between LIDO-1 and LIN-8 in the region deemed minimally necessary for LIN-8-LIN-35 binding (Davison *et al.* 2005). It is possible that LIDO-1 interacts with some of the other genes identified in this screen. A link between LIDO-1 and SynMuv genes is particularly intriguing because *usp-48* mutants, which phenocopy *lido-1* mutants, have previously been shown to genetically interact with the SynMuv machinery (Cui *et al.* 2006).

### nhr-48, a nuclear hormone receptor gene, restricts cellular plasticity

Two other mutants with the Ecta phenotype, *ot880* and *ot881*, were mapped to the same region on the X chromosome, and both contained different missense mutations in the gene *nhr-48* (Figure 7). A genetic complementation cross showed they were alleles of the same gene. In addition, another mutant strain in our collection of Ecta mutants harbored a premature stop codon in the *nhr-48* locus. All *nhr-48* mutant strains are viable and fertile with no obvious pleiotropies. As with the other genes described above, we find that the ectopic expression of the *gcy-5* reporter transgene upon *che-1* expression in *nhr-48* mutants is an accurate reflection of endogenous *gcy-5* transcription since we observed endogenous *gcy-5* induction by smFISH analysis (Figure 9).

*nhr-48* is a member of the family of nuclear hormone receptors (NHR), a class of TFs that contain an N-terminal DNA-binding domain (DBD) and a C-terminal ligand-binding domain (LBD). In *C. elegans*, the NHR gene family has undergone a dramatic expansion, with 284 NHR genes compared to 48 and 21 for humans and flies, respectively (Sluder *et al.* 1999; Maglich *et al.* 2001). While most of these expanded genes have no homologs in humans or other non-nematode species, *nhr-48* is a member of the well-conserved NR1 family of NHRs, which also contain the DAF-12 and NHR-8 gene. This family includes the mammalian Vitamin D Receptor (VDR) and Drosophila Ecdysone Receptor (EcR) (Maglich *et al.* 2001). Despite these similarities, NHR-48 lacks significant conservation in the LBD region, and may not contain a functional ligand binding domain (Lu *et al.* 2016). The only known role for NHR-48 in *C. elegans* is to act as a transcriptional repressor in a subset of pharyngeal gland cells (Ghai *et al.* 2012). *nhr-48* is the only gene for which we could not find any hints in the literature for potential interactions with any of the other genes described in this paper.

### Genes that restrict epidermal cellular fate plasticity are broadly expressed

**Figure 11:**
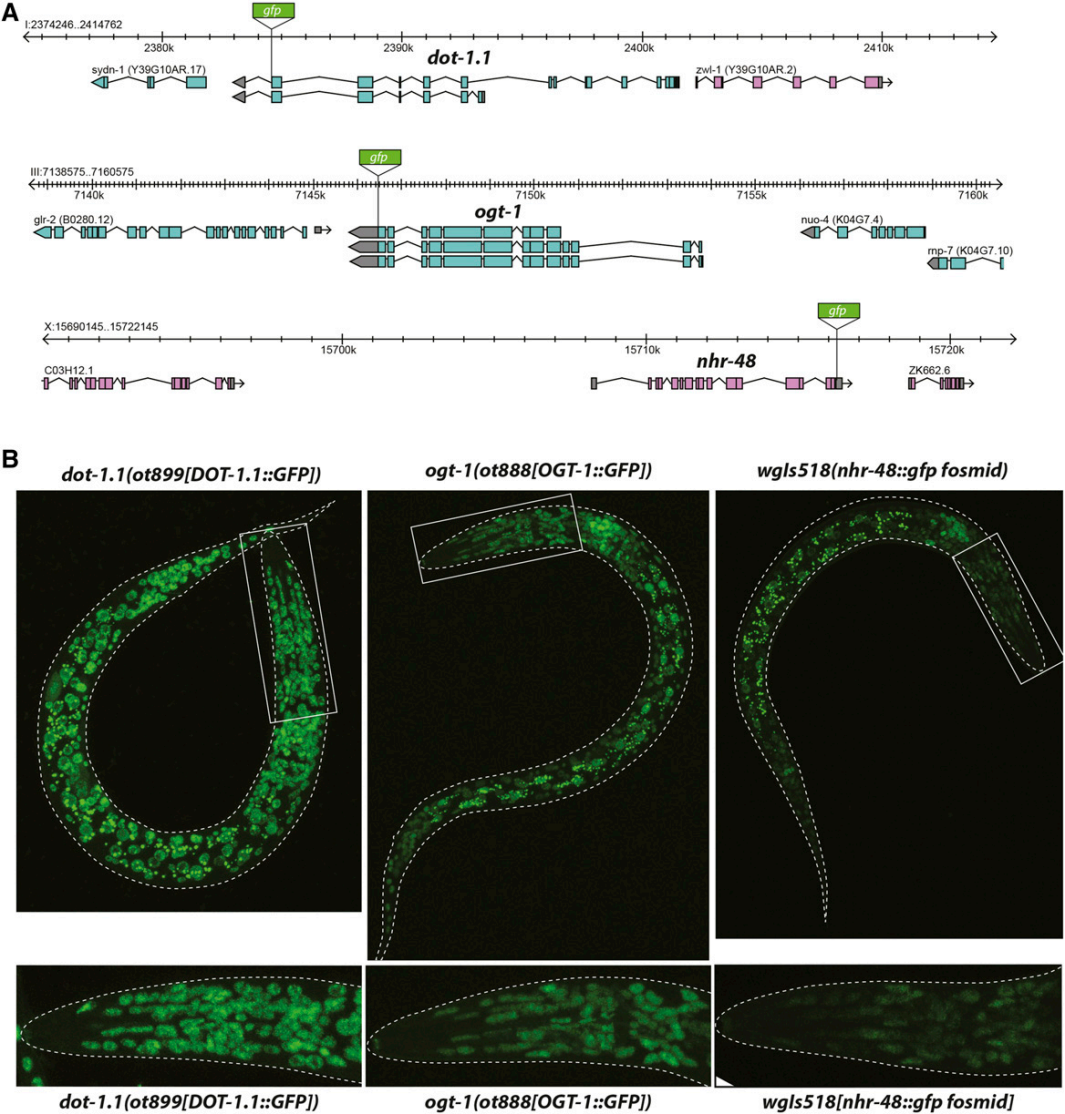
DOT-1.1, OGT-1, and NHR-48 are broadly expressed nuclear proteins. A: Schematic of genomic locus of *dot-1.1*, *ogt-1*, and *nhr-48* indicating where GFP was inserted using CRISPR/Cas9 (*dot-1.1* and *ogt-1*) or fosmid recombineering (*nhr-48*). B: Representative confocal micrographs of L1 animals of each strain. All strains exhibit very broad, if not ubiquitous, expression.

We generated translational C-terminal GFP fusion constructs for a subset of the genes described above. For *dot-1.1* and *ogt-1*, we tagged the endogenous locus with *gfp*, using CRISPR/Cas9-mediate genome engineering, while *nhr-48* expression was examined using a fosmid-based transgene from the TransgeneOme project (Figure 11A)(Sarov *et al.* 2012). In all three cases, each reporter showed very broad if not ubiquitous, nuclear expression throughout development (Figure 11B), as we had also observed with *usp-48* (Figure 4). Similarly, the *pmk-1* gene had previously been shown to be very broadly, if not ubiquitously expressed (Berman *et al.* 2001; Pagano *et al.* 2015). Not only do all these proteins appear to be ubiquitously expressed, but they also all localize to the nucleus (Figure 2, Figure 11). In the case of *pmk-1*, its gene product is reported to translocate to the nucleus upon activation by certain types of stress (Berman *et al.* 2001; Pagano *et al.* 2015).

## Conclusions

We have described here a collection of mutants that permit the ectopically expressed master regulator of ASE neuron identity, *che-1*, to induce ASE-like features in other cell types that are normally refractory to ectopic *che-1* expression. These genes therefore can be considered to normally restrict the plasticity of cellular identity. Several of the genes identified here encode proteins involved in transcription and chromatin modification and based on studies in other organisms, some functional and/or physical links appear to exist between these proteins. We propose that these proteins either directly or indirectly establish a specific chromatin landscape that makes epidermal cell refractory to aberrant gene activation. Hence *che-1* is normally not able to activate its target genes, but can do so upon removal of these genes.

A striking aspect of our findings is that all proteins that we have analyzed here are very broadly, if not ubiquitously expressed nuclear proteins, but the loss of at least one these proteins, USP-48, appears to confer cellular plasticity only to epidermal cells. Using the same *che-1* misexpression assay that we employ here, *ogt-1* mutants were very recently shown to confer cellular plasticity also to germ cells, in which OGT-1 functionally interacts with the chromodomain protein MRG-1 (Hajduskova *et al.* 2019). The plasticity phenotype of *mrg-1* mutants is, in turn, restricted to germ cells (Hajduskova *et al.* 2019). Given that we have previously shown that terminal differentiation of specific neuron types renders cells refractory to ectopic transcription factor-mediated reprogramming, the simplest explanation for the cellular specificity of these plasticity mutants could be that these factors, in spite of their broad expression, have a role in specifying the terminally differentiated state of only specific cell types, such as epidermal cells in the case of *usp-48*. However, none of the mutants that we analyzed appears to have any obvious defects in epidermal cell differentiation, as deduced by overall intact morphology and marker gene expression. Nevertheless, loss of the genes described here must transform the gene regulatory state of epidermal cells in a manner that makes these cells more “plastic”. The normal function of these genes may therefore lie in ensure robustness of the regulatory state of these cells.
